# Attitudes Toward Epilepsy Among Parents of Children With Epilepsy in Southern China

**DOI:** 10.3389/fneur.2020.602000

**Published:** 2021-02-05

**Authors:** Haojun Yang, Yunfang Chi, Ziqing Zhu, Kailing Huang, Lan Xiang, Bo Xiao, Weiting Tang, Li Feng

**Affiliations:** ^1^Department of Neurology, Xiangya Hospital, Central South University, Changsha, China; ^2^Laizhou People's Hospital, Yantai, China; ^3^Department of Neurology, Hunan Provincial People's Hospital, Hunan Normal University, Changsha, China

**Keywords:** attitudes, children with epilepsy, parents, epilepsy, future life

## Abstract

**Purpose:** To evaluate the attitudes toward epilepsy among parents of children with epilepsy (CWE) in China and identify some related factors for future interventions for parents to offer more social support for CWE.

**Method:** The Chinese Public Attitudes Toward Epilepsy (CPATE) scale was administered to 234 parents of CWE and 203 parents of normal children in Xiangya hospital during 2019–2020.

**Results:** The cumulative score of the parents of CWE (26.427 ± 6.688) was significantly lower than that of the normal children group (32.330 ± 7.234, *p* < 0.001). Subanalysis showed more positive attitudes among parents of CWE than the control group (*p* < 0.001) toward education (4.765 ± 1.985 vs. 6.621 ± 2.419), social life (6.556 ± 2.456 vs. 8.010 ± 2.683), marriage (9.586 ± 2.675 vs. 11.025 ± 2.900), and employment (3.876 ± 1.364 vs. 4.5123 ± 1.283). The attitudes toward epilepsy among parents of CWE with seizures in public (27.16 ± 6.66) or during sleep (27.10 ± 6.38) were more negative than those without (25.35 ± 6.62 and 25.08 ± 7.10, respectively) (*p* < 0.05). In addition, female and low income were negatively related to parents' attitudes toward epilepsy.

**Conclusions:** More active policy guidance and adequate social support should be given to parents of children with seizures in public or during sleep to instruct their children to form a positive perception about epilepsy, which is expected to have a positive impact on their social abilities in the future.

## Introduction

Epilepsy is a chronic neurological disorder characterized by unprovoked, recurrent seizures along with abnormal, excessive, or synchronous neuronal activity in the brain. The prevalence rate of epilepsy in children ranges from 3.2‰ to 5.5‰ in developed countries and 3.6‰ to 44‰ in underdeveloped countries (1), which is mostly related to underlying genetic conditions or brain injuries at childbirth, etc. Previous studies have shown that about 30% of children with epilepsy (CWE) still had seizures after antiepileptic drug (AED) therapy (2). These underlying genetics or metabolic conditions, along with poor seizure control, will have negative effects on their cerebral and psychological development. However, it has been suggested that the most debilitated CWE are not necessarily those who have the most frequent seizure attacks, but those who do not get adequate social support (3). High-quality social support has been considered to enhance resilience to stress and even reduce medical morbidity and mortality (4). Parents are undoubtedly the main source of social support (5). Apart from offering financial support for AED, their attitudes toward epilepsy will directly affect the CWE's perception of epilepsy (6) since CWE are too young to get independent and comprehensive views of epilepsy, and cultural restrictions often lead to unsound judgment. Incomplete perception of epilepsy will place them at elevated risk for physiological complications including mental discomfort related to education, marriage, employment, and social life (7, 8). In this case, parents' attitudes toward epilepsy play a vital part in leading CWE to the comprehensive perception of epilepsy.

For CWE and their parents, epilepsy is not only a disease but also a social problem, especially in China. Affected by the imbalanced educational and cultural resources, most families treat seizures as demonic possession, particularly in rural and undeveloped areas (9, 10). Furthermore, under the long-term Family Planning Policy, the family relationship in China is close, which inevitably brings more stress to parents of CWE (11, 12). Our previous work found more severe symptoms of anxiety and depression and poorer sleep quality among parents of CWE, especially in the infants group (13). We hypothesize that mental stress may stem from the pessimism or attitudes toward epilepsy, and the increasing mental stress may in turn aggravate stigma and negative attitudes. This theory highlights the importance of evaluating CWE parents' attitudes toward epilepsy and identifying related factors for positive intervention to promote the efficacy of long-term treatment and reduce the risk of mental disorders.

To date, there has been no comprehensive quantitative study available to evaluate the attitudes toward epilepsy among parents of CWE in China. Therefore, this study aims to comprehensively and quantitatively evaluate the attitudes toward epilepsy among parents of CWE using the c (CPATE) scale and identify some related factors for future interventions for parents to offer high-quality social support for CWE.

## Materials and Methods

### Study Population

This cross-sectional survey was conducted in Xiangya Hospital, Central South University, a 3,500-bed tertiary university hospital with around 100,000 admissions annually in China. Parents whose child aged from just-born to 18 years old was diagnosed with epilepsy in accordance with the International League Against Epilepsy criteria (14) were enrolled *via* convenience sampling at the Department of Pediatrics or Neurology in this study. Children with epilepsy and other chronic diseases (i.e., leukemia, congenital heart disease, diabetes), which could affect the attitudes and mental states of parents, were excluded from this study (15–17). Only the primary caregivers (one participant per family) living with the CWE and taking the main responsibility of caring for the child's physical and mental needs were included in this study. Parents included were also required to have witnessed their children's seizures. Parents of CWE who had epilepsy, alcohol or caffeine addiction, chronic physical disease, or psychiatric illness, as well as during pregnancy or lactation were excluded from this study. Additionally, parents of matching normal children in Hunan province who volunteered to participate formed the control group. Before proceeding to the questionnaire, we clearly explained the aim of this study and got a written consent form. To maintain anonymity, all questionnaires were recorded with a code number instead of names. There were 234 parents of CWE and 203 parents of normal children included from July 2019 to January 2020, who were required to be primary school graduates or above to fully understand the research and effectively fill in the questionnaires.

### Assessment Tools

#### Demographic Data Collection

A detailed general information form was prepared by the researchers for the purpose of this study. The CWE's gender, age, epilepsy course, seizure occasions, and categories of AEDs and their parents' gender, age, residence, education level, monthly income, and fertility status were also collected.

#### Chinese Public Attitudes Toward Epilepsy Scale

The PATE scale, developed by Kheng-Seang Lim from the University of Malaya (18), is a dimensional scale (14 items) used to measure the general and interpersonal views toward people with epilepsy (PWE). There are nine items (Items 1–9) in the general domain to assess the general opinion of PWE and five items (Items 10–14) in the personal domain to evaluate personal relationships with PWE, such as marriage, dating, and employment. The CPATE scale has been verified to have excellent internal consistency (Cronbach's alpha coefficients = 0.853–0.909), with the general domain at 0.909 and the personal domain at 0.853 (reliability) (19). The correlation coefficients between an item and the belonging domain were larger than 0.48, which was higher than the correlation with other domains or the total score (content validity), and the Kaiser–Meyer–Olkin (KMO) value was 0.874 (construct validity) (19). Each item in the CPATE scale was scored by a 5-point Likert Scale, with five being “strongly agree” and 1 being “strongly disagree.” The scores of all positively stated items (Items 2, 5, 10, 11, 14) were reversed. The items in the scale were subcategorized into four social aspects of life, including attitudes toward education (Items 1, 8, and 9), social life (Items 3–5, and 7), marital relationship (Items 6, 10, 11, and 13), and employment (Items 12 and 14). The cumulative score and mean scores in general and personal domains as well as the four social aspects of life were calculated. The cumulative score range of the CPATE scale is 14–60 points, and a higher score represents a more negative attitude.

### Statistical Analysis

All demographic data were analyzed descriptively, with continuous data presented as means and standard variations (mean ± SD) and nominal data presented as frequencies and percentages. Two independent-sample *t*-tests were used to test the score differences between parents of CWE and normal children in two domains and four social aspects. According to the variance analysis and a univariate linear regression model, the mean scores in two domains and four social aspects of life obtained from the parents of CWE group was correlated with demographic characteristic. The remaining explanatory variables that were statistically significant were considered for the multivariate model for each domain and social aspect. A *p*-value < 0.05 was considered statistically significant. Statistical analysis was performed using the SPSS 24.0 package program.

### Ethical Statement

This study was approved by the ethics committees of the Xiangya Hospital of Central South University (No. 201912528) and has been performed in accordance with the ethical standards laid down in the 1964 Declaration of Helsinki. All data were confidential. Participants provided informed consent before participating in the study and were allowed to withdraw from the study at any phase.

## Results

### Demographic Data of Samples

Of the 251 parents of CWE being interviewed, 12 participants refused and five did not complete the questionnaire, so eventually, 234 (93.23%) subjects were included in Group A. A total of 232 parents of normal children were approached, with 19 refusing to be interviewed; 203 (87.5%) agreed to participate as Group B in this study. Table 1 showed the respective proportions of the parents in the CWE group, of which mothers account for the larger one. The mean age of Group B was higher than that of Group A (*p* = 0.025). The analysis of variance showed no difference in gender, residence, education level, income per month, and gender and age of their children.

**Table 1:** Demographic characteristics of the study population.

	**Parents of CWE, *n* (%)**	**Parents of normal children, *n* (%)**	***p*-value**
	234	203	
**Gender**			
Male	76 (32.48)	67 (33.00)	0.907
Female	158 (67.52)	136 (67.00)	
Age	37.56 ± 6.52	38.93 ± 6.16	**0.025**
**Residence**			
Urban	89 (38.03)	96 (47.29)	0.051
Rural	145 (61.97)	107 (52.71)	
**Education level**			
Technical secondary and Junior high school or below	108 (46.15)	81 (39.90)	0.117
Senior high school	79 (33.76)	87 (42.86)	0.051
Undergraduate or above	47 (20.09)	35 (17.24)	0.448
**Income per month**			
<1,000	42 (17.95)	36 (17.73)	0.953
1,000–3,000	59 (25.21)	46 (22.66)	0.533
3,000–5,000	67 (28.63)	58 (28.57)	0.989
>5,000	66 (28.21)	63 (31.04)	0.518
Fertility status^***^			
One kid	73 (31.20)	98 (48.28)	**<0.001**
Two or more kids	161 (68.80)	105 (51.72)	
**Gender of their children**			
Boy	140 (59.83)	107 (52.71)	0.134
Girl	94 (40.17)	96 (47.29)	
**Age of their children (years)**			
<3	64 (27.35)	48 (23.64)	0.238
3–6	49 (20.94)	52 (25.61)	0.248
7–13	66 (28.21)	46 (22.66)	0.185
14–18	55 (23.50)	56 (27.59)	0.328

*p < 0.05,

**p < 0.01,

*** p < 0.001.

Mark of statistical significance is indicated in bold.

*CWE, children with epilepsy*.

### Attitudes Toward Epilepsy Between Parents of Children With Epilepsy and Normal Children Group

**Figure 1:**
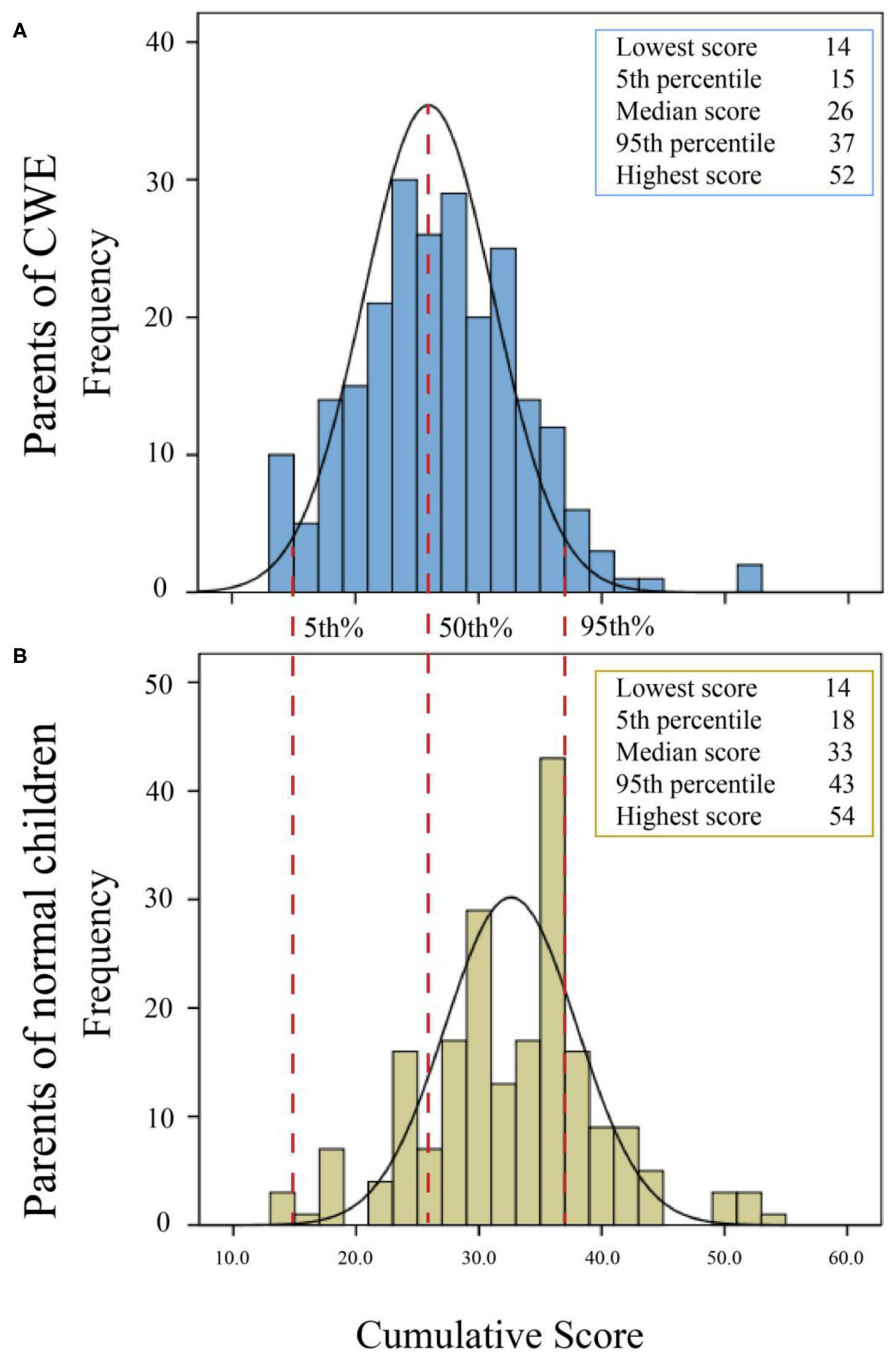
Trend in attitude toward epilepsy presenting in cumulative score histograms of parents of children with epilepsy (CWE) (**A**, blue blocks) and normal children (**B**, yellow blocks). Cumulative score is shown in the x-axis, and the frequency of cumulative score is shown in the y-axis. The black smooth curves represent the normal distribution of the two groups. Three red vertical dotted lines represent the 5th, 50th, and 95th percentiles of distribution of parents of children with epilepsy and how they project on the distribution of parents of normal children.

The cumulative score of the parents of CWE was 26.427 ± 6.688, which was significantly lower than that of the normal children group (32.330 ± 7.234), consistent with the great majority of items (13/14), as shown in Table 2. Response of parents of CWE (ranging from 14 to 52) was obviously better than that of the parents of normal children (ranging from 14 to 54). The median score of responses among parents of CWE placed at 26, whereas the score of parents of normal children was higher, placing at 33 (Figure 1). Additionally, the cumulative scores of 80.30% of parents of normal children were above the 95th percentile of response of parents of CWE, as shown in Figure 1B.

**Table 2:** The means and standard deviations of the scores in each domain and item in parents of children and adolescents with epilepsy and the control group.

**ID**	**Item**	**Mean** **±** **SD**	
		**Parents of children with epilepsy (*n* = 234)**	**Parents of normal children (*n* = 203)**
**General domain**		14.590 ± 4.575	18.902 ± 5.318^***^
1	People with epilepsy should not study in college or university.	1.410 ± 0.695	2.023 ± 0.997^***^
2	People with epilepsy have the same rights as all people.^**r**^	1.645 ± 0.993	2.163 ± 1.033^***^
3	People with epilepsy should be isolated from others.	1.269 ± 0.516	1.818 ± 0.797^***^
4	People with epilepsy should not participate in social activities.	1.393 ± 0.628	1.872 ± 0.798^***^
5	I will not mind being seen in the company with someone known to have epilepsy.^**r**^	2.470 ± 1.856	2.404 ± 1.841
6	People with epilepsy should not marry.	1.624 ± 0.799	2.108 ± 0.819^***^
7	I would stay away from a friend if I knew she/he had epilepsy.	1.423 ± 0.590	1.916 ± 0.984^***^
8	People with epilepsy should study in a special school.	1.761 ± 0.955	2.325 ± 1.109^***^
9	Schools should not place children with epilepsy in regular classrooms.	1.594 ± 0.880	2.271 ± 1.076^***^
**Personal domain**		11.838 ± 3.125	13.429 ± 3.032^***^
10	I would date someone even though he/she has epilepsy.^**r**^	2.692 ± 0.962	2.966 ± 1.041^**^
11	I would marry someone with epilepsy, even though he/she has epilepsy.^**r**^	2.731 ± 0.922	3.030 ± 0.933^***^
12	I feel uncomfortable working with someone who has epilepsy.	1.714 ± 0.729	1.975 ± 0.754^***^
13	I will advise my family members against marrying someone with epilepsy.	2.538 ± 1.040	2.921 ± 1.031^***^
14	If I am an employer, I would give equal employment opportunities to someone with epilepsy.^**r**^	2.162 ± 0.980	2.537 ± 1.059^***^
**Cumulative score**		26.427 ± 6.688	32.330 ± 7.234^***^

** p < 0.01,

*** p < 0.001.

r *These items were reversely scored*.

### Attitudes Toward Four Social Aspects of Life in People With Epilepsy Between Parents of Children With Epilepsy and Normal Children Group

**Figure 2:**
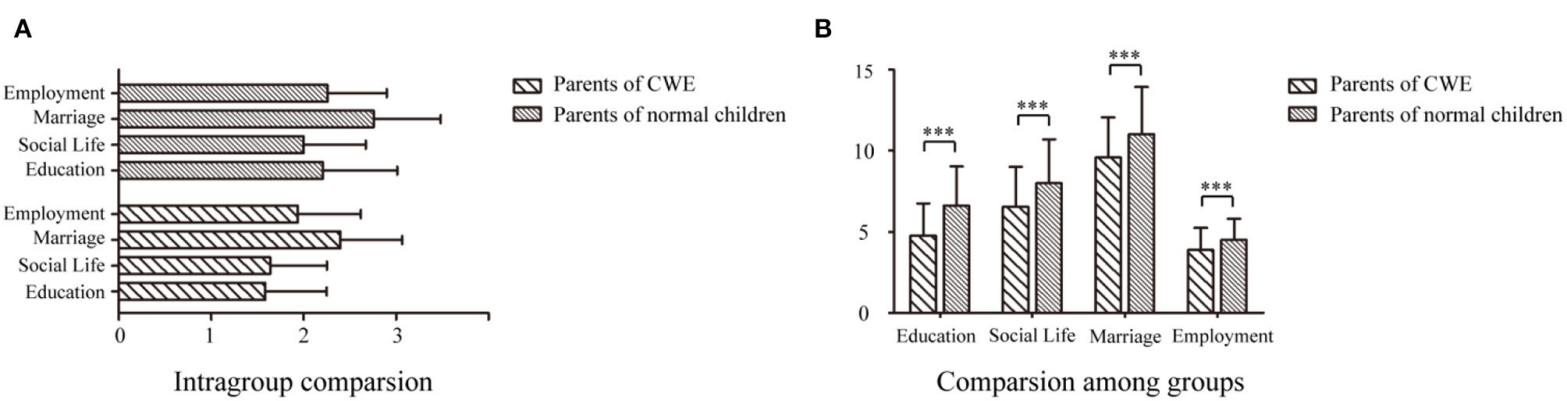
Attitude toward the four social aspects of life in people with epilepsy among parents of children and adolescents with epilepsy and the control group. **(A)** Intragroup comparison: Mean scores per item of attitudes toward the four social aspects of life in people with epilepsy among parents of children and adolescents with epilepsy and the control group, respectively. **(B)** Comparison among groups: Attitudes toward four social aspects of life in people with epilepsy parents of children and adolescents with epilepsy and the control group. Statistical significance (*p* < 0.001) is marked with ***.

Subanalysis showed that attitudes toward various social aspects of life in people with epilepsy among parents of CWE were much more positive than those of the control group (Figure 2), including attitudes toward education (4.765 ± 1.985 vs. 6.621 ± 2.419, *p* < 0.001), social life (6.556 ± 2.456 vs. 8.010 ± 2.683, *p* < 0.001), marriage (9.586 ± 2.675 vs. 11.025 ± 2.900, *p* < 0.001), and employment (3.876 ± 1.364 vs. 4.5123 ± 1.283, *p* < 0.001), as shown in Figure 2B. In the group of CWE, intragroup comparison showed the mean scores were marriage (2.396 ± 0.669), employment (1.938 ± 0.682), social life (1.639 ± 0.614), and education (1.588 ± 0.662), in a descending order. However, the control group showed some difference with the mean score of education being the third highest (2.207 ± 0.806) and social life being the lowest (2.002 ± 0.671), as shown in Figure 2A.

### Factors Related to Parental Attitudes Toward Epilepsy

The results of univariate analysis indicated a history of seizures in public or during sleep of CWE and the gender, income, fertility status of their parents were correlated with the parents' attitudes (Table 3), which could be applied for further multiple analysis. The multiple linear regression suggested five predictors that could explain 11.1% of the cumulative score (*R*^2^ = 0.130, *F* = 6.803), including gender (β = 0.126, *p* = 0.048), income (β = −0.132, *p* = 0.043), fertility status (β = 0.136, *p* = 0.031), history of seizures during sleep (β = −0.125, *p* = 0.046), and AED categories (β = 0.183, *p* = 0.004).

**Table 3:** Mean scores by domains, social aspects of life, and demographic characteristics among parents of children with epilepsy.

**Variable**	**Groups (n)**	**Cumulative score**	**General domain**	**Personal domain**	**Education**	**Social Life**	**Marriage**	**Employment**
Age^**a**^		0.028	0.035	0.010	0.078	−0.041	−0.004	0.054
		**Mean** **±** **SD**	**Mean** **±** **SD**	**Mean** **±** **SD**	**Mean** **±** **SD**	**Mean** **±** **SD**	**Mean** **±** **SD**	**Mean** **±** **SD**
Gender	Male	24.89 ± 6.78	13.80 ± 4.35	11.09 ± 3.33	4.66 ± 1.99	6.10 ± 2.19	8.81 ± 2.78	3.84 ± 1.49
	Female	**27.16** **±** 6.54^*^	14.96 ± 4.64	**12.20** **±** 2.96^*^	4.82 ± 1.99	6.77 ± 2.55	**9.96** **±** 2.55^**^	3.89 ± 1.30
Residence	Urban	25.69 ± 7.06	14.01 ± 5.02	11.68 ± 3.07	4.20 ± 1.62	6.48 ± 3.10	9.64 ± 2.68	3.66 ± 1.20
	Rural	26.88 ± 6.43	14.94 ± 4.26	11.93 ± 3.16	**5.11** **±** 2.11^***^	6.60 ± 1.97	9.55 ± 2.68	4.01 ± 1.44
Education level	Technical secondary and Junior high school or below	26.72 ± 6.72	14.86 ± 4.35	11.87 ± 3.32	5.04 ± 2.14	6.58 ± 1.97	9.43 ± 2.74	**4.06** **±** 1.48^**^
	Senior high school	27.03 ± 6.09	14.89 ± 4.30	12.14 ± 2.85	4.55 ± 1.71	6.89 ± 2.06	9.80 ± 2.48	3.94 ± 1.19
	Undergraduate or above	24.81 ± 7.34	13.50 ± 5.37	11.31 ± 3.06	4.44 ± 1.96	5.98 ± 3.69	9.62 ± 2.85	3.33 ± 1.21^**t**^
Income per month	<1,000	**29.53** **±** 6.14^**^	**16.43** **±** 4.12^*^	13.09 ± 3.00	**5.57** **±** 2.16^**^	**7.09** **±** 1.91^**^	**10.51** **±** 2.49^***^	**4.36** **±** 1.64^*^
	1,000–3,000	26.40 ± 6.28^**t**^	14.42 ± 4.32^**t**^	11.98 ± 3.23	4.54 ± 1.83^**t**^	6.66 ± 1.96	9.58 ± 2.58	4.00 ± 1.26
	3,000–5,000	24.41 ± 6.39^**t**^	13.41 ± 4.12^**t**^	11.00 ± 3.16	4.39 ± 1.85^**t**^	6.05 ± 1.89^**t**^	8.72 ± 2.57^**t**^	3.72 ± 1.28^**t**^
	>5,000	25.91 ± 6.98^**t**^	14.39 ± 5.16^**t**^	11.52 ± 2.86	4.64 ± 1.95^**t**^	6.54 ± 3.44	9.68 ± 2.79	3.54 ± 1.18^**t**^
Gender of their children	Boy	26.77 ± 6.61	14.65 ± 4.21	12.12 ± 3.24	4.80 ± 1.98	6.57 ± 2.02	9.76 ± 2.74	3.98 ± 1.46
	Girl	25.91 ± 6.79	14.50 ± 5.09	11.41 ± 2.90	4.71 ± 2.01	6.53 ± 2.99	9.33 ± 2.57	3.71 ± 1.19
Age of their children	<3	26.22 ± 8.16	14.85 ± 5.70	11.38 ± 3.64	4.69 ± 2.13	6.86 ± 3.40	9.35 ± 3.11	3.68 ± 1.39
	3–6	26.54 ± 5.43	14.51 ± 3.96	12.03 ± 2.58	4.73 ± 1.89	6.43 ± 1.76	9.68 ± 2.36	4.00 ± 1.37
	7–13	26.27 ± 5.72	14.20 ± 3.76	12.07 ± 2.84	4.61 ± 1.79	6.38 ± 1.84	9.87 ± 2.48	3.83 ± 1.27
	14–18	26.82 ± 6.60	14.80 ± 4.34	12.02 ± 3.09	5.07 ± 2.11	6.45 ± 2.06	9.47 ± 2.52	4.11 ± 1.42
Only a kid or not	Yes	24.68 ± 6.75	13.38 ± 4.89	11.30 ± 3.13	4.12 ± 1.80	6.34 ± 3.25	9.37 ± 2.67	3.45 ± 1.16
	No	**27.22** **±** 6.53^**^	**15.14** **±** 4.33^**^	12.08 ± 3.10	**5.06** **±** 2.00^**^	6.65 ± 1.99	9.68 ± 2.68	**4.07** **±** 1.41^**^
Course of epilepsy	<1 year	26.75 ± 7.11	14.82 ± 4.59	11.92 ± 3.48	4.94 ± 2.02^**t**^	6.56 ± 2.16	9.61 ± 2.79	3.94 ± 1.44
	1–3 years	25.72 ± 6.68	14.18 ± 5.05	11.54 ± 3.10	**4.11** **±** 1.54^**^	6.88 ± 3.36	9.49 ± 2.72	3.61 ± 1.21
	>3 years	26.62 ± 6.35	14.66 ± 4.26	11.96 ± 2.84	5.04 ± 2.13^**t**^	6.34 ± 1.95	9.63 ± 2.56	4.00 ± 1.38
Having seizures during	Yes	**27.10** **±** 6.38^*^	14.97 ± 4.63	**12.13** **±** 2.89^*^	4.86 ± 1.87	6.72 ± 2.64	**9.88** **±** 2.48^*^	3.93 ± 1.28
sleep	No	25.08 ± 7.10	13.82 ± 4.39	11.26 ± 3.50	4.58 ± 2.19	6.23 ± 2.02	9.00 ± 2.96	3.77 ± 1.52
Having seizures in public	Yes	**27.16** **±** 6.66^*^	**15.15** **±** 4.79^*^	11.94 ± 3.02	**5.00** **±** 1.98^*^	**6.81** **±** 2.73^*^	9.54 ± 2.53	**4.06** **±** 1.37^**^
	No	25.35 ± 6.62	13.77 ± 4.13	11.68 ± 3.28	4.42 ± 1.95	6.18 ± 1.94	9.65 ± 2.89	3.60 ± 1.31

* p < 0.05,

** p < 0.01,

*** p < 0.001. Mark of statistical significance is indicated in bold.

t These groups were significantly different compared to the group with */**/*** in the same domain or aspect.

a *Based on Pearson's correlations*.

### Factors Related to Chinese Public Attitudes Toward Epilepsy Scores by Domains

Univariate analysis showed that more negative attitudes toward epilepsy in the general domain were found in parents of CWE with low income, multiple children, and a history of seizures in public of their sick child (Table 3). Additionally, more negative attitudes appeared in the personal domain among maternal parents of CWE whose children had seizures during sleep. The multiple linear regression found that two predictors explained 6.2% of the variance (*R*^2^ = 0.070, *F* = 8.690, *p* < 0.001) in the general domain and three predictors explained 6.7% of the variance (*R*^2^ = 0.079, *F* = 6.613, *p* < 0.001) in the personal domain. We found that AED categories were associated with attitudes toward epilepsy in both general (β = 0.196, *p* = 0.002) and personal (β = 0.156, *p* = 0.015) domains. Fertility status (β = 0.170, *p* = 0.008) was an independent factor associated with attitudes toward epilepsy in the general domain (Table 4). Also, income (β = −0.187, *p* = 0.004) and gender of their children (β = −0.125, *p* = 0.050) were identified as independent factors associated with attitudes toward epilepsy in the personal domain (Table 4). Neither the general nor the personal domain of attitudes toward epilepsy among parents of CWE was independently associated with age, residence, education level of the parents, and the age, course, and seizure locations of CWE.

**Table 4:** Results of multivariate analysis in domains and aspects of life among parents of children with epilepsy.

**Variable**	***B***	**SE**	**β**	***t***	***p***	***R***	***R*^**2**^**	**Adjusted *R*^**2**^**
**Cumulative score**								
Constant	23.09	2.92		7.91	0.000	0.360	0.130	0.111
Gender	1.801	0.905	0.126	1.990	0.048			
Income	−0.776	0.382	−0.132	−2.033	0.043			
Fertility status	1.961	0.904	0.136	2.168	0.031			
Having seizures during sleep	−1.775	0.885	−0.125	−2.006	0.046			
AED categories	0.896	0.309	0.183	2.898	0.004			
**General domain**								
Constant	10.735	1.132		9.481	0.000	0.265	0.070	0.062
Fertility status	1.676	0.626	0.170	2.679	0.008			
AED categories	0.657	0.213	0.196	3.085	0.002			
**Personal domain**								
Constant	13.733	0.827		16.609	0.000	0.282	0.079	0.067
Income	−0.515	0.176	−0.187	−2.921	0.004			
Gender of their children	−0.794	0.403	−0.125	−1.969	0.049			
AED categories	0.358	0.146	0.156	2.446	0.015			
**Education**								
Constant	2.295	0.571		4.020	0.000	0.281	0.079	0.071
Residence	0.741	0.265	0.182	2.798	0.006			
Fertility status	0.752	0.278	0.176	2.709	0.007			
**Social Life**								
Constant	6.330	0.745		8.498	0.000	0.180	0.032	0.024
Gender	0.665	0.339	0.127	1.963	0.049			
Having seizures in public	−0.632	0.323	−0.127	−1.957	0.048			
**Marriage**								
Constant	8.847	0.776		11.402	0.000	0.259	0.067	0.059
Gender	1.182	0.363	0.207	3.261	0.001			
Having seizures during sleep	−0.931	0.360	−0.164	−2.586	0.010			
**Employment**								
Constant	4.538	0.537		8.454	0.000	0.328	0.108	0.092
Income	−0.207	0.078	−0.172	−2.653	0.009			
Gender of their children	−0.357	0.174	−0.129	−2.050	0.041			
Fertility status	0.510	0.187	0.173	2.731	0.007			
Having seizures in public	−0.344	0.178	−0.124	−1.928	0.049			

*AED, antiepileptic drug*.

### Factors Related to Chinese Public Attitudes Toward Epilepsy Scores by Aspects

As shown in Table 3, more negative attitudes toward marriage were found among mothers of CWE (9.96 ± 2.55) than fathers (8.81 ± 2.78, *p* = 0.002). Registered rural parents of CWE (5.11 ± 2.11) showed more negative attitudes than registered urban parents of CWE (4.20 ± 1.62, *p* < 0.001) toward education. The lower education level of parents of CWE, the more negative attitudes toward employment (*p* = 0.003) were seen. Parents with monthly income <1,000 yuan showed significantly negative attitudes in all aspects of education, social life, marriage, and employment (*p* < 0.05). More negative attitudes were found in marriage among parents of children with seizures during sleep (9.88 ± 2.48) than those without (9.00 ± 2.96, *p* = 0.018). Besides, parents of children with seizure attacks in public had more negative attitudes in the aspect of education (5.00 ± 1.98), social life (6.81 ± 2.73), and employment (4.06 ± 1.37) than those without (4.42 ± 1.95, *p* = 0.028; 6.18 ± 1.94, *p* = 0.049; 3.60 ± 1.31, *p* = 0.010, respectively).

The multiple linear regression found two predictors that could explain 7.1% of the variance (*R*^2^ = 0.079, *F* = 9.881, *p* < 0.001) in the aspect of education; another two same predictors explained 2.4 and 5.9% of the variance in the aspect of social life (*R*^2^ = 0.032, *F* = 3.853, *p* = 0.023) and marriage (*R*^2^ = 0.067, *F* = 8.295, *p* < 0.001), respectively, and four predictors explained 9.2% of the variance in the aspect of employment (*R*^2^ = 0.108, *F* = 6.916, *p* < 0.001).

## Discussion

To better understand the CWE parents' attitudes toward epilepsy, the simplified CPATE scale was conducted among parents of CWE in Hunan province for the first time. This study provides detailed characterization of the stigma toward epilepsy among parents of CWE. Compared to the control group, the attitudes toward epilepsy among parents of CWE were more positive. However, they tended to hold negative attitudes if seizures of their children occur in public or during sleep.

Parents of CWE showed much more positive attitudes toward epilepsy, which was plausible by their deepening knowledge about this disease. They tended to have a more professional understanding of epilepsy because of their long-term care and health education. Therefore, they had a relatively rational attitude toward epilepsy. Previous studies have shown that awareness raising about epilepsy and its etiology could improve people's attitudes (20, 21). Similarly, Chinese medical staff demonstrated significantly positive attitudes toward people with epilepsy compared to the general population, indicating that health education could be a new public intervention to reduce disease stigma (22, 23). Additionally, empathy might be another explanation for these positive attitudes from parents of CWE. Empathy is thought to be a unique ability of humans to feel, understand, and share the emotional state of others (24), making it possible to resonate with others' positive and negative feelings alike, more commonly seen in persons working in helping professions, such as doctors, therapists, and caregivers (25, 26). Parents' emotional identification of their children's suffering drove them to have a relatively positive attitude toward epilepsy (27, 28). So they tended to have a higher compliance with the hope of helping their children to control seizures and avoid psychosocial problems faced in the course of marriage, education, and employment. Hopeful attitudes of getting cured with treatment and satisfaction with present treatment were also found in mothers of newly diagnosed pediatric cancer patients and parents of youth with juvenile rheumatic diseases (JRDs) (29, 30). Accordingly, it is not hard to understand the relatively more positive attitudes toward epilepsy among parents of CWE, which express their inner expectations of better future and social life with the lowest epilepsy influence for their children.

The dilemma of epilepsy is associated not only with seizure control but also with a range of psychosocial problems faced in the course of marriage, education, and employment (31). Patients with epilepsy often experience marriage problems, such as low marital prospects, poor marital outcomes, reduced marital satisfaction, as well as increased rates of divorce (32). Previous studies found that PWE were 3.7 times more likely to remain single than healthy controls (33), and the divorce rate was twice more than that of the general population (34). Beside the higher infertility rate of PWE, these problems mostly resulted from negative attitudes toward epilepsy for stigma in the context of marriage (35). In this study, both parents of CWE and normal children showed significant negative attitudes toward epilepsy in the aspect of marriage, which was consistent with previous studies (36, 37). Interestingly, we found that parents of CWE with nocturnal seizures were particularly pessimistic about marriage mainly because nocturnal seizures might be psychological burdens on their spouses (36). Worries about seizures appearing in the next generation are another reason for their pessimism about marriage, especially when the partner considers epilepsy a totally genetic disease unilaterally (38). Additionally, we found that attitudes toward education, employment, and social life among parents of CWE with seizure attacks in public were more negative than those without. Affected by traditional views that epilepsy is caused by evil spirits and can be transmitted from patients, CWE and their parents often suffer from severe stigma in China, especially in rural and undeveloped areas (39). People are often worried about being attacked by CWE especially when they accompanied with some cognitive deficits or mental disorders or being contaminated by the patient with epilepsy (13, 40).

Studies have shown that symptoms of anxiety and depression are related to the occurrence of seizures in public among adolescents with epilepsy, leading to a substantial negative impact on educational achievement and employability in adulthood (6, 41, 42). Parents may attribute their children's unwillingness to socialize and get education and employment to concerns of irregular or unpredictable seizures in the daytime. Because of stigma and shame, they also do not want their children to be seen by the public when seizure attacks, which is bound to affect their children's future life. And children's perception of epilepsy is largely influenced by their parents' attitudes. According to the social learning theory, children internalize their parents' attitudes or beliefs for seeking parental acceptance, fostering intergenerational correspondence in attitudes (43). It has been proven that in teenagers with JRD and their parents, parents' perceptions regarding JRD could affect their child's attitudes and ultimate child adjustment outcomes (30). In this case, children will experience the same feelings of shame toward seizure attacks in public as their parents do. Therefore, positive intervention for parents to cultivate comprehensive attitudes toward epilepsy to reduce stigma and shame, such as active policy guidance, education and support for parental self-care, and guidance for engaging effective social supports (21, 44, 45), can promote children's perception of epilepsy and reduce the risk of mental disorders.

In addition, we found female and low income were negatively related to parents' attitudes toward epilepsy. Previous studies have shown that the maternal emotion statuses and quality of life in CWE seemed much worse than the paternal's (46), which is consistent with our findings. Diversity and fluctuation of circulating ovarian hormones and neurotransmitter may explain the gender differences in attitudes toward epilepsy in our study (47). Furthermore, epilepsy, as one of the most common chronic neurological diseases, implies high medical expenses for long-term medication, which is undoubtedly a heavy financial burden on low-income families. Previous studies has found that low income and related economic pressure might result in lack of education and poor health knowledge, contributing to a negative attitude toward disease (48).

Some limitations of this study must be acknowledged. The data of a single-center study cannot represent the general attitudes toward epilepsy of the parents of CWE in Southern China, and the sample size of participants in this study is relatively small. Moreover, we hope to conduct multicenter studies and recruit more parents of CWE for verification in the future. Besides, more attention should be paid to the identification of related factors and the observation of the impact of intervention on seizure control, cognition, marriage, and employment in patients of children with childhood-onset epilepsy.

## Conclusion

This is the first study to quantitatively evaluate the attitudes toward epilepsy among parents of CWE in China and identify significant predictive factors for future interventions for parents of CWE. The attitudes toward epilepsy among parents of CWE were more positive than those of parents of normal children probably due to the deepening understanding of epilepsy and empathy. In addition, the attitudes toward epilepsy among parents of CWE with seizures in public or during sleep were more negative than those without. We also found that female and low income were negatively related to parents' attitudes toward epilepsy. In light of this information, more positive intervention, such as active policy guidance, education and support for parental self-care, and guidance for engaging effective social supports, should be paid to parents of children with seizures to instruct their children in forming positive perception about epilepsy, which is expected to have a positive impact on their social abilities in the future.

## Data Availability Statement

The raw data supporting the conclusions of this article will be made available by the authors, without undue reservation.

## Ethics Statement

The studies involving human participants were reviewed and approved by The ethics committee of Xiangya Hospital of Central South University. Written informed consent to participate in this study was provided by the participants' legal guardian/next of kin.

## Author Contributions

HY, WT, BX, and LF conceived of the analysis. HY, YC, ZZ, KH, LX, and BX contributed to the data collection and analysis. HY and ZZ wrote the first draft of the manuscript. KH, LX, WT, BX, and LF provided critical feedback on the first draft. HY, WT, and LF managed the production process. All authors read and approved the final manuscript.

## Conflict of Interest

The authors declare that the research was conducted in the absence of any commercial or financial relationships that could be construed as a potential conflict of interest.
